# Defectors, not norm violators, are punished by third-parties

**DOI:** 10.1098/rsbl.2014.0388

**Published:** 2014-07

**Authors:** Jonathan Bone, Antonio S. Silva, Nichola J. Raihani

**Affiliations:** 1 CoMPLEX, University College London, London WC1E 6BT, UK; 2 Department of Anthropology, University College London, London WC1E 6BT, UK; 3 Department of Genetics, Evolution and Environment, University College London, London WC1E 6BT, UK

**Keywords:** cooperation, punishment, social, norms, cheats, sanction

## Abstract

Punishment of defectors and cooperators is prevalent when their behaviour deviates from the social norm. Why atypical behaviour is more likely to be punished than typical behaviour remains unclear. One possible proximate explanation is that individuals simply dislike norm violators. However, an alternative possibility exists: individuals may be more likely to punish atypical behaviour, because the cost of punishment generally increases with the number of individuals that are punished. We used a public goods game with third-party punishment to test whether punishment of defectors was reduced when defecting was typical, as predicted if punishment is responsive to norm violation. The cost of punishment was fixed, regardless of the number of players punished, meaning that it was not more costly to punish typical, relative to atypical, behaviour. Under these conditions, atypical behaviour was not punished more often than typical behaviour. In fact, most punishment was targeted at defectors, irrespective of whether defecting was typical or atypical. We suggest that the reduced punishment of defectors when they are common might often be explained in terms of the costs to the punisher, rather than responses to norm violators.

## 1. Introduction

Humans have a strong tendency to conform to social norms of behaviour [1–3]. Conformity can be an adaptive response to uncertainty regarding the appropriate behaviour in a specific context: by observing how others behave in that setting, individuals might be better able to infer what behaviour is successful [4] and what is likely to be approved or disapproved by others [2]. Compliance with social norms has been argued to underpin the existence of large-scale cooperation in human societies [5]. Specifically, humans are thought to conform to a social norm of conditional cooperation, which is enforced by punishment of those who violate the norm [6]. Thus, defectors should be less likely to be punished, or be punished less severely, when they are in the majority rather than the minority. Some evidence exists to support this idea. For example, third-party punishment of defectors in a Prisoner's Dilemma game is more severe when the partner cooperates than when both players defect [7]. Similarly, individuals in public goods game (PGG) are more likely to be punished the more their contribution deviates from the group average [8,9].

While these findings have been interpreted as evidence that punishment is motivated by a dislike of norm deviants, we suggest an important alternative explanation: individuals are more likely to punish atypical defectors because this is by definition cheaper than punishing defectors when defection is common. In most previous studies, this explanation for the punishment of atypical behaviour has not been ruled out, because the costs of punishment increase with the number of individuals that are punished (e.g. [8,9]). We used a PGG with third-party punishment and experimentally manipulated the number of cooperators and defectors to test whether punishment is aimed specifically at norm deviants or, more generally, at defectors, when there is no additional cost to punishing the majority. We also measured the third-parties desire to exclude individuals from a subsequent PGG game as an indicator of social rejection.

## 2. Material and methods

Data were collected in March 2014. We recruited 1050 subjects (664 males, 380 females and six unspecified) for our experiment using the online labour market, Amazon Mechanical Turk (www.mturk.com). Subjects were all based in the USA. We used a PGG to test whether punishment was motivated by the norm violation in this setting. Players were randomly allocated to the role of player 1–4 (*n* = 840) or to the role of player 5 (*n* = 210). Players 1–4 played a PGG, while player 5 was an observer who could choose to punish any or all of the four PGG players after they made their contributions. After the game, all subjects were required to fill in a questionnaire to provide demographic information (electronic supplementary material, table S2).

In the PGG, players 1–4 were allocated an investment token and informed that they could invest this in a ‘public investment opportunity’ or a ‘private investment opportunity’. Public investments yielded $0.20 to the investor and $0.20 to each of the other players. Conversely, private investments yielded $0.30 to the investor and nothing to the other players. Thus, investing publicly was equivalent to cooperating while investing privately was equivalent to defecting, or free-riding, in standard PGGs. players 1–4 were assigned to groups ex-post [10] to create two conditions: the ‘typical defector’ condition (three defectors and one cooperator) and the ‘atypical defector’ condition (three cooperators and one defector).

Player 5 observed the decisions of players 1–4, either in the typical defector condition (*n* = 102) or the atypical defector condition (*n* = 108). Player 5 was allocated $1.05 and could choose whether to pay a fixed cost ($0.05) to reduce the earnings of any of the other players by $0.15. Player 5 could punish one, two, three or all four of the PGG players for the same fixed cost of $0.05; thus, the increasing costs associated with punishing more than one player were removed in this game.

Subsequent to the punishment decision, player 5 rated each PGG player on a seven point scale as to how much they would like to play a subsequent investment game with that player (similar to [9,11]). This answer provided a measure of social rejection. The majority of ratings were either one or seven (proportion = 0.68 ± 0.2) so we re-categorized ratings into a binary variable for analysis. Ratings less than four were set as 1 (indicating desire to avoid the player in question) and ratings of four or more were set as 0 (indicating indifference, or preference for the player in question).

Data were analysed using R v. 3.02 [12]. Using two generalized linear mixed models (GLMMs), we measured the probability that a player would be (i) punished and (ii) socially rejected by player 5 according to how they behaved (cooperator/defector) and whether or not the behaviour violated the social norm in that setting. We additionally controlled for the effects of age and gender on player 5's propensity to punish. We employed a multi-model inference approach [13]. Input variables were standardized [14]. We estimated the importance and model-averaged coefficients of parameters using a set of models with the highest support (within 2AICc units of the top model) [15]. We only present the parameter estimates from the top models (see the electronic supplementary material for further details).

## 3. Results

**Figure 1.**
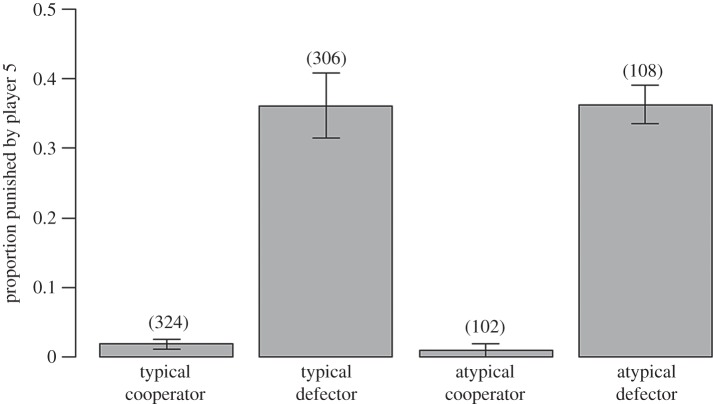
The proportion of PGG players who were punished by player 5, according to their PGG decision and whether this violated the descriptive norm. Sample sizes for each condition are indicated in parentheses. Error bars show standard errors.

In general, typical and atypical behaviours were equally likely to be punished (proportion of typical behaviour punished = 0.17 ± 0.02; versus atypical = 0.22 ± 0.04; table 1). In addition, defectors were just as likely to be punished whether their behaviour was typical (0.36 ± 0.03) or atypical (0.36 ± 0.05; table 1 and figure 1). Similarly, cooperators were rarely punished, regardless of whether their behaviour was typical (0.02 ± 0.01) or atypical (0.01 ± 0.01; table 1 and figure 1). Cooperators were never singled out for costly punishment and only faced punishment when all members of their group were also punished (on three occasions). Furthermore, when player 5 invested to punish defectors, they always punished all defectors in the group rather than singling one individual out for punishment. Punishment was linked to gender, with male players being more likely to punish than females (proportion of individuals that were punished by males = 0.22 ± 0.02; versus females = 0.12 ± 0.02; table 1).

**Table 1.** Estimates, unconditional standard errors, confidence intervals and relative importance for parameters included in the top models explaining whether PGG players were punished by player 5.

parameter	estimate	unconditional s.e.	confidence interval	relative importance
intercept	−4.35	0.56	(−5.45, −3.25)	
PGG decision (cooperate/defect)	5.98	1.02	(3.96, 8.00)	1.00
player 5 gender (female/male)	2.19	0.49	(1.22, 3.16)	1.00
player 5 age	−0.25	0.42	(−1.08, −0.59)	0.30

The results for social rejection mirrored the punishment investment decisions above: cooperative individuals were preferred as partners over defectors for a hypothetical subsequent PGG, regardless of whether cooperative behaviour was typical or atypical (proportion defectors rejected *typical* = 0.84 ± 0.03; *atypical* = 0.80 ± 0.05). Although, players appear to reject atypical cooperators slightly more often than typical cooperators, the confidence intervals for the interaction term just crossed zero, meaning that the evidence for this effect is weak (cooperators rejected *typical* = 0.3 ± 0.01; *atypical* = 0.5 ± 0.02; table 2; electronic supplementary material, figure S1).

**Table 2.** Estimates, unconditional standard errors, confidence intervals and relative importance for parameters included in the top models explaining whether PGG players were socially rejected by player 5.

parameter	estimate	unconditional s.e.	confidence interval	relative importance
intercept	−1.31	0.49	(−2.27, −0.34)	
PGG decision (cooperate/defect)	9.45	0.99	(7.51, 11.39)	1
violated the social norm (no/yes)	0.56	0.81	(−1.02, 2.14)	0.8
violated the social norm × PGG decision	−2.21	1.59	(−5.32, 0.90)	0.8
player 5 gender (female/male)	0.74	0.56	(−0.36, 1.82)	0.62

## 4. Discussion

Previous studies have suggested that punishment might be proximately driven by the desire to harm individuals that violate social norms. However, these studies have typically not controlled for the possibility that paying to harm norm violators is less costly than paying to harm conformers, because the costs of punishing typically scale with the number of individuals that are punished [7,9,16]. Here, we removed this scaling effect of punishment by allowing individuals to pay a fixed cost to punish any or all of the PGG players. Under these conditions, individuals directed almost all punishment towards defectors regardless of whether defecting was the norm. These results contradict the prediction that defectors are less likely to be punished when they are typical [7] and suggest that defectors are probably viewed negatively regardless of their prevalence in the population. In other studies, rare defectors may receive more punishment than common defectors because this is less costly to the punisher. It is possible that defectors were punished regardless of their prevalence, because individuals did not make punishment decisions based on the events in the game but instead on a pre-existing perception of defection as a norm violation formed from their experience in the ‘real world’. However, previous studies in the same cultural group (US-based subjects) have shown that individuals' behaviour is sensitive to similar social norm manipulations that occur within the confines of the game setting [9,11].

We found very little evidence for antisocial punishment in this setting, even when cooperators were in the minority. This contradicts previous findings, which have shown that excessively generous individuals are singled out for punishment, even though their behaviour ostensibly benefits the individuals who punish them [9]. The rarity of antisocial punishment in our current study may be because many of the motives proposed to underpin antisocial punishment were absent in our setting. Most previous studies of antisocial punishment have shown that it comes from individuals within the group, rather than third-parties, suggesting that antisocial punishment reflects competition for status within groups [17]. For example, antisocial punishment might occur in retaliation for punishment received (or expected to be received) from cooperators [17,18]. Alternatively, since individuals are often chosen as partners based on their cooperativeness relative to others [19–21], defectors might punish cooperators because cooperators ‘raise the bar’, making defectors look bad in comparison [18,22]. In the absence of these motives, we found no evidence to suggest that norm deviants were more likely to be punished by third-parties. Our measures of social rejection, however, did hint that atypical cooperators were slightly less likely to be preferred for subsequent hypothetical interactions, when player 5 would then be in the group with this individual. This tendency, although weak, supports previous work showing that excessively helpful, cooperative or moralistic individuals might be viewed negatively rather than positively by others in their social group [9,11,23,24].

To summarize, third-party punishers targeted defectors, rather than norm violators in this setting. We suggest that decreased punishment of defectors when common might reflect the increased cost of punishing. Although, atypical cooperators were infrequently punished in this setting, they were slightly less preferred for subsequent interactions. Thus, the lack of antisocial third-party punishment in our setting might reflect the fact that punishers were not in competition for status with cooperators [17]. Punishment of cooperative norm violators might be more common from fellow group members, rather than third-parties.

## Funding statement

This study was funded by a Royal Society University Research Fellowship to N.R.

## Supplementary Material

Electronic supplementary materials

## Supplementary Material

Data
