# Supplementary material for: Defectors, not norm violators, are punished by third-parties
Source: Biol Lett. 2014 Jul;10(7):20140388. doi: 10.1098/rsbl.2014.0388 (PMC4126631; doi:10.1098/rsbl.2014.0388)
Supplement: Electronic supplementary materials [file rsbl20140388supp1.docx]

**Electronic supplementary materials**

**Table of contents**

|  |  |  |
| --- | --- | --- |
| 1 | Table S1 | p. 2 |
| 2 | Table S2 | p. 3 |
| 3 | Figure S1 | p. 4 |
| 4 | Supplementary methods  Experimental protocol  Statistical methods | p. 5  p. 5 – 6 |
| 5 | R code used to fit generalised linear mixed models | p. 7 |
| 6 | References |  |

| Treatment | Player | PGG decision | Payoff | Norm violated |
| --- | --- | --- | --- | --- |
| Atypical cooperator | Player 1 | Defect | $0.60 | No |
|  | Player 2 | Defect | $0.60 | No |
|  | Player 3 | Defect | $0.60 | No |
|  | Player 4 | Cooperate | $0.90 | Yes |
|  | Player 5 | NA | $1.05 | NA |
| Atypical defector | Player 1 | Cooperate | $0.50 | No |
|  | Player 2 | Cooperate | $0.50 | No |
|  | Player 3 | Cooperate | $0.50 | No |
|  | Player 4 | Defect | $0.20 | Yes |
|  | Player 5 | NA | $1.05 | NA |

**Table S1.** The Public Goods Game (PGG) decision, payoff received and whether or not this PG game decision violated the descriptive norm for Players 1 – 5 in the atypical cooperator and atypical defector treatment.

| **Parameter** | **Players 1 - 4** | **Player 5** |
| --- | --- | --- |
| Age | Mean = 29.36 ± 0.3 | Mean = 30.14 ± 0.67 |
|  | Median = 27 | Median = 27.5 |
|  | IQR = 24 – 33 | IQR = 23 – 33.25 |
|  | Range = 18 - 66 | Range = 18 -68 |
|  | Prefer not to disclose = 6 | Prefer not to disclose = 2 |
| Education level (n) | Some High School = 8 | Some High School = 1 |
|  | High School Graduate = 76 | High School Graduate = 14 |
|  | Some College, no degree = 282 | Some College, no degree = 64 |
|  | Associates Degree = 64 | Associates Degree = 20 |
|  | Bachelors Degree = 322 | Bachelors Degree = 79 |
|  | Graduate Degree = 82 | Graduate Degree = 32 |
|  | Prefer not to disclose = 6 | Prefer not to disclose = 0 |
| Gender (n) | Females = 313 | Females = 67 |
|  | Males = 521 | Males = 143 |
|  | Prefer not to disclose = 6 | Prefer not to disclose = 0 |
| Annual income (n) | Less than $12,500 = 79 | Less than $12,500 = 20 |
|  | $12,500 - $24,999 = 105 | $12,500 - $24,999 = 37 |
|  | $25,000 - $37,499 = 142 | $25,000 - $37,499 = 24 |
|  | $37,500 - $49,999 = 101 | $37,500 - $49,999 = 33 |
|  | $50,000 - $62,499 = 101 | $50,000 - $62,499 = 30 |
|  | $62,500 - $74,999 = 88 | $62,500 - $74,999 = 15 |
|  | $75,000 - $87,499 = 42 | $75,000 - $87,499 = 6 |
|  | $87,500 - $99,999 = 39 | $87,500 - $99,999 = 16 |
|  | $100,00 or more = 86 | $100,00 or more = 21 |
|  | Prefer not to disclose = 57 | Prefer not to disclose = 8 |

**Table S2.** Information on mean and median values (where appropriate) and sample sizes for demographic information for Players 1 – 4 and Player 5.


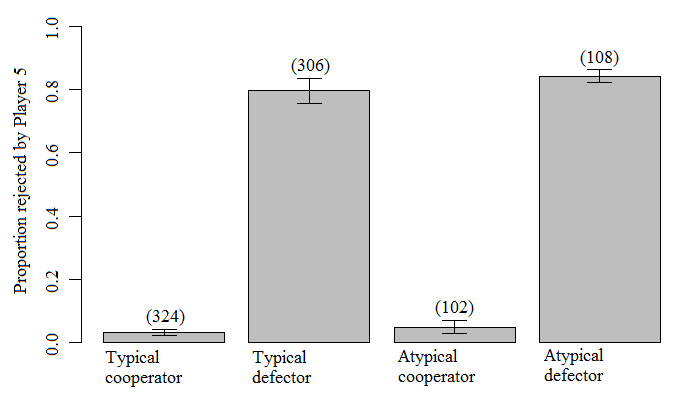


**Figure S1**. The proportion of PGG players who were socially rejected by Player 5, according to their PGG decision and whether this violated the descriptive norm. Sample sizes for each condition are indicated in parentheses. Error bars show standard errors.

**Supplementary Results**

An equal proportion of Player 5s invested in costly punishment in both the typical defector (0.36 ± 0.05) and the atypical defector treatment (0.36 ± 0.05). In both treatments, costly punishment was focussed on defectors rather than cooperators. Thus, antisocial punishment was uncommon in this setting (proportion of defectors punished = ± 0.36 ± 0.02; proportion cooperators punished = 0.02 ± 0.01).

**Supplementary Methods**

All subjects were paid a show-up fee of $0.30 on top of a bonus based on their payoff in the game. All Players (1 – 5) were required to answer nine comprehension questions. Players were required to answer all comprehension questions correctly to take part in the game. Each subject was only allowed to participate in the experiment once. Subjects recruited through AMT are identified by a unique 14-digit worker ID rather than by their names. To prevent subjects from repeatedly participating in the experiment, only one entry was allowed per unique ID and subjects were prevented from repeatedly accessing the external survey website from the same IP address. Participants remained anonymous throughout the experiment and were informed that their worker ID would not be revealed to their partner. Subjects were then redirected to an external survey website ([https://opinio.ucl.ac.uk](https://opinio.ucl.ac.uk/)) to take part in the experiment.

Player 5's initial endowment was chosen so as to exceed the largest possible payoff of any of the PGG players to rule out disadvantageous inequity aversion as a motive for punishing defectors (e.g. [1,2]).

At the end of the experiment players were asked on a seven point scale (1 = not at all, to 7 = very much) how confident they were that the other players in their group were real people. It is important that players were confident that they were playing real people because punishment decisions have been shown to be different when people play human players compared to computer players [3]. We repeated all analyses excluding players that gave an answer of less than four (proportion answering < 4 = 0.46 ± 0.02) to the question above but found that this did not change the key findings of the study. Although the proportion of players that gave an answer of less than four was relatively high, it is likely that by asking this question we increased their suspicion that they weren’t really playing real people.

**Statistical methods**

All GLMMs produced had a binomial error distribution and logit link and were fitted using the lme4 package [4]. GLMMs allow repeated measures to be fitted as random terms, thus controlling for their effects on the distribution of the data. We ran two models with our data. In model (i), we asked whether each of the Players 1 - 4 (n = 840) was punished by Player 5. The response term was set as '1' if the player was punished and '0' if the player was not punished. In model (ii), we asked whether the player was socially rejected by Player 5. Again, the response term was set as '1' if a player was rejected (preference score of < 4) and '0' if a player was not rejected (preference score of > 4). For both analyses, we included the following explanatory variables in the model: 'PGG decision' (cooperate / free-ride), ‘violated norm’ (no / yes) and the two-way interaction between these variables. We also controlled for players' age and gender (male / female). For each model, 'Player 5 ID' was included as the random term.

For each analysis we initially generated a global model. Following specification of the global model, all binary explanatory variables were centred by subtracting the mean. Continuous explanatory variables were centred and standardised by dividing by two standard deviations [5]. We used the package MuMIn [6] to generate all possible submodels from this initial global model. A subset of top models was identified by taking the best model) and any models within 2AICc units of the best model [7,8]. Using this subset of models, we computed the average parameter estimates for each term included in the subset of models, as well as the relative importance of the term. Importance is calculated by summing the Akaike weights of all models that include the term in question and can be thought of as the probability that the given term is a component of the best model [9].

**R code used to fit generalised linear mixed models**

#Load data and packages

library(MuMIn)

library(arm)

library(lme4)

data<-read.csv("Data.csv", header=T)

#Model 1

glmer(Punished~ PG.game.decision*Social.norm.violated+Player.5.gender+Player.5.age

+(1| Player.5.id),data=data,family="binomial")->global.model

stdz.model <- standardize(global.model,standardize.y = FALSE)

model.set <- dredge(stdz.model)

top.models <- get.models(model.set, subset = delta<2)#gave 2 models

model.avg(top.models)->m1

summary(m1)

confint(m1)

#Model 2

glmer(Rejection.binary~PG.game.decision*Social.norm.violated+Player.5.gender+Player.5.age

+(1|Player.5.id),data=data,family="binomial")->global.model

stdz.model <- standardize(global.model,standardize.y = FALSE)

model.set <- dredge(stdz.model)

top.models <- get.models(model.set, subset = delta<2)#gave 2 models

model.avg(top.models)->m2

summary(m2)

confint(m2)

**References**

1. Johnson, T., Dawes, C. T., Fowler, J. H., McElreath, R. & Smirnov, O. 2009 The role of egalitarian motives in altruistic punishment. *Econ. Lett.* **102**, 192–194.

2. Raihani, N. J. & McAuliffe, K. 2012 Human punishment is motivated by inequity aversion, not a desire for reciprocity. *Biol. Lett.* , 18–21.

3. Sanfey, A. G., Rilling, J. K., Aronson, J. A., Nystrom, L. E. & Cohen, J. D. 2003 The neural basis of economic decision-making in the Ultimatum Game. *Science (80-. ).* **300**, 1755–1758.

4. Bates, D., Maechler, M. & Bolker, B. 2011 lme4: Linear mixed-effects models using S4 classes. *Compr. R Arch. Netw.* , 0.

5. Gelman, A. 2008 Scaling regression inputs by dividing by two standard deviations. *Stat. Med.* **27**, 2865–73.

6. Barton, K. 2013 {MuMIn}: multi-model inference, {R} package version 1.9.5.

7. Burnham, K. P. & Anderson, D. R. 2004 *Model Selection and Multimodel Inference: A Practical Information-Theoretic Approach*. Springer.

8. Grueber, C. E., Nakagawa, S., Laws, R. J. & Jamieson, I. G. 2011 Multimodel inference in ecology and evolution: challenges and solutions. *J. Evol. Biol.* **24**, 699–711.

9. Johnson, J. B. & Omland, K. S. 2004 Model selection in ecology and evolution. *Trends Ecol. Evol.* **19**, 101–8.
